# Wearable Wireless Functional Near-Infrared Spectroscopy System for Cognitive Activity Monitoring

**DOI:** 10.3390/bios15020092

**Published:** 2025-02-06

**Authors:** Mauro Victorio, James Dieffenderfer, Tanner Songkakul, Josh Willeke, Alper Bozkurt, Vladimir A. Pozdin

**Affiliations:** 1 Department of Electrical and Computer Engineering, Florida International University, Miami, FL 33174, USA; 2 Department of Electrical and Computer Engineering, North Carolina State University, Raleigh, NC 27695, USAaybozkur@ncsu.edu (A.B.); 3 Department of Engineering Physics, Rose Hulman Institute of Technology, Terre Haute, IN 47803, USA; willekjw@rose-hulman.edu; 4 Department of Mechanical and Materials Engineering, Florida International University, Miami, FL 33174, USA

**Keywords:** near-infrared spectroscopy (NIRS), functional NIRS (fNIRS), wearable devices, biosensors, brain activity, cognitive monitoring

## Abstract

From learning environments to battlefields to marketing teams, the desire to measure cognition and cognitive fatigue in real time has been a grand challenge in optimizing human performance. Near-infrared spectroscopy (NIRS) is an effective optical technique for measuring changes in subdermal hemodynamics, and it has been championed as a more practical method for monitoring brain function compared to MRI. This study reports on an innovative functional NIRS (fNIRS) sensor that integrates the entire system into a compact and wearable device, enabling long-term monitoring of patients. The device provides unrestricted mobility to the user with a Bluetooth connection for settings configuration and data transmission. A connected device, such as a smartphone or laptop equipped with the appropriate interface software, collects raw data, then stores and generates real-time analyses. Tests confirm the sensor is sensitive to oxy- and deoxy-hemoglobin changes on the forehead region, which indicate neuronal activity and provide information for brain activity monitoring studies.

## 1. Introduction

Individuals suffering from cognitive fatigue experience slower and delayed reaction times as compared to a rested state [1]. In addition to the personal effects of cognitive exhaustion [2,3,4], cognitive fatigue has a significant impact on society, increasing workplace and highway accidents and fatalities [5,6,7]. Some studies have even shown that exhaustion can manifest in behaviors similar to those of intoxication [4,8]. However, cognitive fatigue is not as apparent as intoxication and can be difficult to identify and quantify.

Over the past few decades, functional magnetic resonance imaging (fMRI) has been used to image volumetric electrical activity in the brain under various stimuli and workloads to map the functional regions and assess the characteristics of cognitive fatigue [9,10]. While it has been foundational in understanding the role of fatigue on brain function, fMRI equipment is not portable. Recently, functional near-infrared spectroscopy (fNIRS) has been used to monitor brain activity by measuring changes in oxygenated and deoxygenated hemoglobin in reflection geometry. While fNIRS restricts measures to the surface of the brain, with penetration up to a mere 10–30 mm [11], the correlation with fMRI is clear [11]. Relying on decades of fMRI research, fNIRS is emerging as a portable alternative to brain activity monitoring.

Full-scalp fNIRS brain activity mapping is a promising technique for portable cognitive fatigue detection for drivers [12], pilots [13,14], critical workers, or even to improve training or interventions [15]. However, real-world full-scalp measurements have limitations due to cumbersome hardware, optical losses from hair, and user adherence [16,17]. Furthermore, mapping of the entire brain may not be necessary for a cognitive assessment; comparative studies have shown that most activity happens in the prefrontal cortex (PFC) [18,19,20]. The PFC is responsible for working memory, mental imagery, and higher cognitive functions [21,22,23]. In addition, the blood flow in the PFC is 20 to 30% higher than the average brain blood flow, with activity levels up to 50% higher compared to other brain regions [24]. Furthermore, the PFC is easily accessible in the vast majority of individuals in various occupational scenarios. These factors make PFC well suited to the portable monitoring of brain activity using fNIRS for point-of-care assessments of cognitive fatigue.

Miniaturization brings the advantages of availability and imperceptible sensing to fNIRS, which enable the possibility of unencumbered testing. However, miniaturized devices suffer from lower signal fidelity due to reduced signal conditioning, reduced functionality and sensor count, and additional error and noise from unencumbered testing. Miniaturized counterparts will not match the precision and accuracy of benchtop analogs, but their shortcomings are overshadowed by their potential use cases and the promise that large datasets and AI will enable real-time diagnostics anywhere. Toward the development of a portable and wearable fNIRS platform, we report on the design and validation of a bandage-sized wireless system with high sensitivity that can be adapted to various geometries. The firmware development aimed to enable flexible usage, along with resilience, to overcome usage error and drift. The hardware design for fNIRS has been optimized for adoption with next-generation conformable electronics. The presented system has been validated using occlusion, breath-holding, and cognitive workload tests to demonstrate the potential of wearable fNIRS for the future of AI-driven algorithm development.

## 2. Materials and Methods

**Figure 1:**
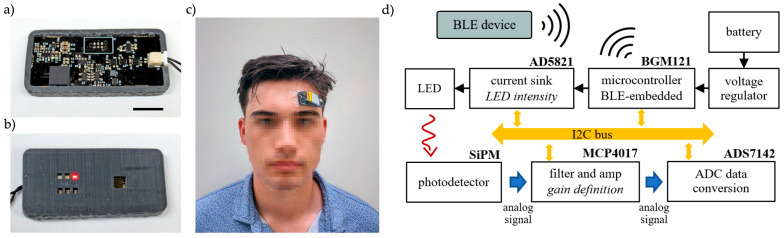
Developed wearable fNIRS system. (**a**) Top view of the device components, (**b**) skin-facing view with the flexible shroud applied, (**c**) prototype device on a participant, attached using 3M Tegaderm to fix both the sensor and the battery, and (**d**) device block diagram. The scale bar is 10 mm.

The key to wearable design is miniaturization and flexibility. Miniaturization is critical in rigid printed circuit boards (PCBs), as large footprints cannot conform to the curvature of the human body, leading to signal losses and user discomfort. PCB design can be improved further by replacing the rigid board with an ultra-thin polymeric circuit board, which can be realized with a single-layer, double-sided design. With these considerations, a proof-of-concept wearable fNIRS sensor was designed as a 2-layer circuit board to place the photodetector and LEDs on the skin-facing side and the rest of the electronics on the other side (see Figure 1). Commercial-off-the-shelf (COTS) LEDs and a photodetector were used in this work; however, we have previously demonstrated a method to realize flexible inorganic optical elements [25], which can provide conformable optics in the 2-layer design. A flexible 3D-printed shroud was used to improve the interface between the planar circuit board and the curved forehead. In addition to increasing user comfort, the shroud blocks background light and improves signal quality. The shroud was printed using a 3D printer (Form3, Formlabs Inc., Somerville, MA, USA) using Flexible 80A resin. The shroud was designed to match the height of the thickest optical component, which in our design, are the LEDs. The entire completed device was coated with parylene C for biocompatibility [26].

### 2.1. Hardware

The sensing platform was based on a commercially available 32-bit microcontroller (BGM121, Silicon Laboratories Inc., Ausitn, TX, USA) that controls the optode activation, LED intensity, signal gain, ADC readings, and BLE communication. The parameters for light source selection and intensity, gain levels, and sampling frequency were defined by auto-calibration routines or entered manually by the user through a BLE protocol. 

The platform contained multiple source–detector pairs to investigate oxygen changes with optode separation, to accommodate various device placement locations on the body, and to provide resilience to variations in users’ placement of the device. LEDs were arranged in three banks at 20.2 mm, 17.7 mm, and 15.2 mm source–detector distances; wavelength values of 660 nm (APT2012SRCPRV, Kingbright Inc., City of Industry, CA, USA) and 840 nm (15406085BA300, Würth Elektronik Inc., Waldenburg, Germany) were used in each bank. A Silicon Photomultiplier (SiPM) (MicroFC 30035, On Semi Inc., Scottsdale, AZ, USA) with an active area of 9 mm^2^ was used for detection of the light signals.

The communication between the microcontroller and peripheral devices was made over an I2C bus, the LED enabling was managed by using MOSFETs (NTLUD3A50PZ, On Semi Inc., Scottsdale, AZ, USA) controlled by GPIO ports from the microcontroller, and the light intensity was defined by a DAC current driver (AD5821, Analog Devices Inc., Wilmington, MA, USA). Detection readings were transduced to voltage measurements over a 100 Ω resistor in series with the photodetector. Prior to analog-to-digital conversion, the voltage signal would pass through a buffer, a low-pass filter with a 7 kHz cut-off frequency, and a variable gain stage. Signal processing circuitry was implemented using low-noise op amps (MCP6001R, Microchip Inc., Chandler, AZ, USA). The filter stage was a second order Sallen–Key. A non-inverting configuration was used for amplification; variable gain was achieved by utilizing a digital potentiometer (MCP4017, Microchip Inc., Chandler, AZ, USA) in the feedback loop. The microcontroller would then retrieve the photodetector measurements from the ADC (ADS7142, Texas Instruments Inc., Dallas, TX, USA) over the I2C bus and store it in memory before a timed Bluetooth low energy (BLE) transmission to the external device. The device block diagram is summarized in Figure 1d.

The sensing platform was conceived to be compact and comfortable, reaching wearability requirements needed for a device that must be used for long periods of time. All the functionalities, including the optode configuration, activation, data collection, conversion, and BLE transmission, were processed in a 19 × 44 mm circuit board. The entire system, including the battery, was secured to the user’s forehead using a medical film dressing (3M Tegaderm, St. Paul, MN, USA). The device was first adhered to the center of the dressing, ensuring at least a 1-cm adhesive border. Then, the dressing was applied to the skin. The platform utilizes a lithium-ion 3.7 V (nominal value) battery. A 500 mAh battery can provide 50 h of operation between charges under continuous operation with a sampling rate of 10 Hz. For a specific use case, continuous measurements may not be necessary, allowing the DeepSleep function to significantly extend battery life.

#### 2.1.1. LED Characterization

**Figure 2:**
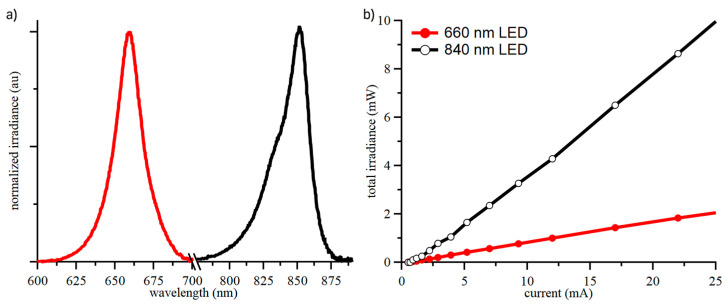
Spectral output (**a**) and total irradiated power (**b**) of the LEDs used for fNIRS sensing and operated at drive currents an order of magnitude below the safety limits for irradiated power. The different colors are subliminal messaging for red and NIR distinction. (**a**) contains data for two devices.

The calculation of both oxy- and deoxy-hemoglobin changes required readings at two different wavelengths, and the chosen values were 660 nm and 840 nm. LED spectral output and power were characterized using a spectrophotometer (LR1, Aseq Instruments Inc., Vancouver, BC, Canada) and an integrating sphere (IS6, StellarNet Inc., Tampa, FL, USA) to determine the hemoglobin optical constants and safety limits for the LED drive current based on IEC62471 (see Supplementary Materials), as shown in Figure 2.

#### 2.1.2. Optical System Optimization

Significant optical losses and signal corruption occur at the body–device interface. To improve the blocking of ambient light, the photodetector was fitted with an optical filter (LEE 026 Bright Red-LEE Filters, Burbank, CA, USA). In addition, black silicone was applied around the sides of the photodetector and between the PCB and the shroud to reduce stray light from reaching the detector. In addition, polydimethylsiloxane (Sylgard 184 (Dow, Midland, MI, USA) at a 10:1 ratio and cured at 80 °C for 1 h) was used to fill the empty spaces around the LEDs and photodetector and create direct contact with the skin.

The effectiveness of the optical coupling was evaluated on the same device before and after fabrication. The coupling was evaluated on the wrist to reduce signal variation due to cortical hemodynamics. Measurements were collected with a fixed hardware gain and variable LED drive current. From the collected 30-s time series, the AC and DC components of the hemodynamic response were extracted using MATLAB (MathWorks Inc., Natick, MA, USA) and normalized per total LED irradiance in mW. We termed this value as the coupling factor (CF).

Effective coupling of light from the LED to the skin and from the skin to the photodetector was critical to achieve high data fidelity and low power operation. The addition of a clear, compressible polymer to fill the air gaps between the device–skin interface significantly improved the CF in our device, as seen in Table 1. The improvement in CF scaled with optode separation and achieved an approximately 10× improvement at 20.2 mm of separation. The CF improvement for the 660 nm LED coupling was higher than the 840 nm LED, potentially due to the shallower penetration depth of the shorter wavelength. The difference between the DC and AC coupling factors was attributed to the vascular structure at the placement site. Consistent improvement in both CFs demonstrated that the polymer-capped optical package would achieve lower power consumption, particularly with large optode separations.

### 2.2. Software and Interface

Firmware was developed to manage fNIRS signal acquisition with a BLE connection. The device initially enters an idle state with BLE advertising when powered on. When a BLE connection is established, advertising stops, the measurements of light absorption start, and data are sent over a dedicated UUID. To control the device parameters during operation, a second two-byte UUID was created to support user commands for sampling frequency, optode activation, light intensity, photodetector gain, and self-calibration routines (see Supplementary Materials).

#### 2.2.1. Optode Operation

**Figure 3:**
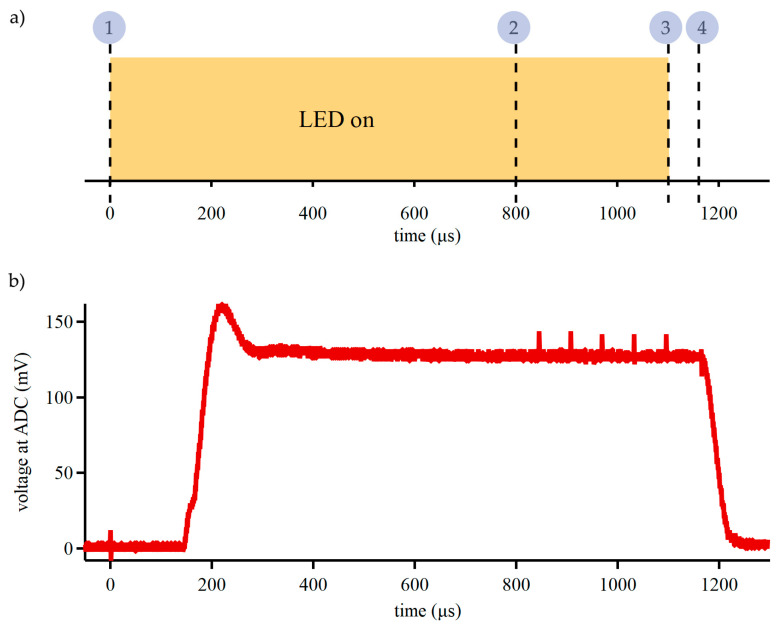
Optical system timing for low power and precision measurements. (**a**) At first, (1) the current driver is enabled, and LED MOSFET is switched on; (2) after an 800 µs delay, ADC readings start; (3) after the readings, the current driver is disabled and the LED MOSFET is switched off; and (4) the drive current and gain values for the next LED are written. (**b**) The voltage at the ADC recorded by an oscilloscope during the optical system’s activation shows delayed LED activation and signal stabilization. The optical signal stabilizes within 800 µs.

Accurate calculations of hemodynamic changes in our device required reliable optical measurements. A high-sensitivity photodetector (~10^6^ A/W) was utilized in our design to minimize the LED drive current and to support large optode separation in future works. The timing of optical component activations was optimized to minimize power consumption and overcome the time constant of the system. Before signal acquisition started and immediately after the previous LED deactivation, the value for the next LED drive current was written to the DAC register and the gain for the signal processing circuit was set on the digital potentiometer. The sequence for signal acquisition is shown in Figure 3a. ADC readings of the signal from the photodetector were requested 800 µs after the LED MOSFET was activated to allow for the stabilization of the LED output, DAC output, and SiPM. After the ADC readings, the current sink was disabled and MOSFET was switched OFF.

The stability and precision of optical signal transduction was evaluated by comparing 5 sequential readings taken at 16.67 kHz (60 µs apart). This assessment task was conducted with the device placed on a reflective phantom running a custom firmware routine. The results from 5 sequential readings were compared using a paired two-sided *t*-test with a 1% significance level (see the Supplementary Materials for details). For ADC measurements initiated 800 µs after the current driver and MOSFET, there was no statistical difference between the sequential readings, which indicates that the optical system was in a steady state. A period of ~1000 µs is the fastest reliable sampling time achievable with our device for a single optode pair. The optode signal stabilization was the main limitation to the sampling speed of our device; however, hemodynamic changes are significantly slower than the fundamental limit of our device.

#### 2.2.2. Data Processing

All ADC data were filtered using an ideal 2 Hz low-pass filter prior to conversion to the hemoglobin concentration. Changes in the oxy- and deoxy-hemoglobin concentration, Δ*C_HbO_* and Δ*C_Hbb_*, were calculated from the measured intensity of the reflected light using the modified Beer–Lambert law (MBBL) [27,28,29,30]. Changes in concentration were calculated using:

(1) $$\left[\begin{array}{c} ∆C_{HbO}\\ ∆C_{Hb} \end{array}\right]={\left(d\cdot {\left(\left[\begin{array}{cc} \varepsilon_{HbO_{\lambda_{1}}} & \varepsilon_{HbO_{\lambda_{2}}}\\ \varepsilon_{Hb_{\lambda_{1}}} & \varepsilon_{Hb_{\lambda_{2}}} \end{array}\right]\times \left[\begin{array}{cc} DPF_{\lambda_{1}} & 0\\ 0 & DPF_{\lambda_{2}} \end{array}\right]\right)}^{T}\right)}^{-1}\times \left[\begin{array}{c} ∆OD_{\lambda_{1}}\\ ∆OD_{\lambda_{2}} \end{array}\right]$$

where *d* is the distance between the source and the detector, *ε_HbOλi_* and *ε_Hbλi_* are the extinction coefficient for both oxy- and deoxy-hemoglobin at each wavelength, *OD* is the optical density, and *DPF* is the differential pathlength factor, defined as follows [27,30,31]:

(2) $$DPF_{\lambda i}=\frac{1}{2}{\left(\frac{3{\mu^{\prime }}_{s,\lambda i}}{\mu_{a,\lambda i}}\right)}^{1/2}\left[1-\frac{1}{1+d\cdot {\left(3{\mu^{\prime }}_{s,\lambda i}\mu_{a,\lambda i}\right)}^{1/2}}\right]\;$$

where *μ_a,λi_* and *μ′_s,λi_* are the absorption and reduced scattering coefficients for each wavelength *i*, respectively. Coefficients used in calculations were taken from the literature [32,33] and are summarized in the Supplementary Materials.

Optical density changes, Δ*OD_λi,_* were calculated in relation to a baseline, which enabled calculations that were independent of the intensity emitted by the LEDs. Once the intensity was selected, an initial acquisition interval was used as a baseline and then the changes were taken as a ratio to the baseline. This approach also supports computational updates to overcome changes in the LED light intensity or ambient lighting for use in a dynamic environment. The ADC readings were sent over the BLE to a custom GUI interface, and changes in concentration computations were conducted off-chip to reduce the device’s power consumption and to visualize the data in real time.

In addition to custom software for real-time data analysis, offline computation was carried out using the Homer3 toolbox available in MATLAB [34]. For Homer3 data processing, the raw ADC data were imported and corrected for artifacts. First, motion artifacts were removed using hmrR_MotionArtifact routine with *tMotion = 0.5*, *tMask = 1.0, STDEVthresh = 50,* and *AMPthresh = 50.* Afterwards, correlation-based signal improvement (CBSI), principal component analysis (PCA), Savitz–Golay (SG) correction, and wavelet filtering were applied to improve the quality of the hemoglobin concentration changes data. The SG correction was used with *p = 0.99* and *FrameSize_sec = 10.* Motion wavelet correction was used with *IQR = 1.5*.

### 2.3. Human Subject Testing

Testing was divided into two phases: validation and two human subject experiments. For fundamental validation, an occlusion test was performed with the sensor on the wrist and a sphygmomanometer cuff placed around the upper arm. We collected data from a 5-min interval of seated rest prior to occlusion to establish a baseline. The cuff was inflated to 200 mmHg, which is above the systolic pressure, to induce arterial occlusion [35,36]. In the second validation test, the sensor was placed on the prefrontal cortex while the participant was instructed to hold their breath to record hemodynamic changes at different optode distances [37]. The participant performed two 30-s breath-holding intervals, preceded by a relaxed inhale (to avoid hyperventilation), with a 60-s resting period between intervals [38,39]. The sensor configuration settings for both validation tests—occlusion and breath-holding—were determined using the autocalibration function. This process defined the LED intensity current values and amplification gain for signal detection, with the resulting values provided in Table S2 in the Supplementary Materials. All measurements were collected at 10 Hz. Changes in hemoglobin concentration were filtered using a 5-s moving mean.

To assess the capabilities of the developed sensor to measure cognitive activity, participants were recruited to perform arithmetic operations with the sensor placed on the left prefrontal cortex. For this study, participants performed a series of three-digit and two-digit additions, similar to other fNIRS study [40]. Our work utilized a block protocol design to induce a cyclic pattern of mental effort, alternating mental math with rest and alternating the mental math difficulty (three-digit and two-digit). For the duration of testing, the participants remained seated and followed the prompts on a computer monitor. Before and after mental activities, long resting periods were used to collect baseline and recovery data. Each mental activity task was a 2-min block of arithmetic, followed by a 2-min block of rest. The study was conducted in two groups. The first group of 5 participants had 10 min of rest before and after the arithmetic tasks, with 5 cycles of three-digit and two-digit additions. The second group of 5 participants had 20 min of rest before and 30 min of rest after the arithmetic tasks, with 3 cycles of additions. The entire testing protocol was developed on a custom graphical interface in MATLAB for exact timing and to remove user distractions. During the long resting periods, participants watched relaxing videos, and during the 2-min blocks of rest, a countdown timer was displayed on the screen. Additional details of the study are in the Supplementary Materials. During the study, research staff supervised the participants and noted their performance and behavior.

On-body evaluations of the fNIRS devices were performed in compliance with an IRB-approved protocol (FIU 22–0506). Subjects gave written informed consent before participating in the study.

## 3. Results

### 3.1. Device Validation Studies

**Figure 4:**
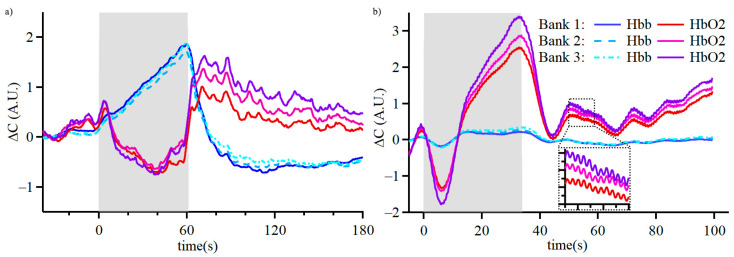
Validation study of hemodynamic responses measured using the wearable fNIRS device. Real-time measurements of (**a**) arterial occlusion with the sensor on the wrist and (**b**) breath-holding with the sensor on the left prefrontal cortex. The inset shows the PPG waveform for the heart rate calculations.

The developed wearable device was initially assessed for tissue oxygenation measurements in an arterial occlusion test. During arterial occlusion, blood flow in and out of the appendage is impeded, and the supply of oxygen is consumed. In the process, the oxy-hemoglobin concentration decreases and deoxy-hemoglobin increases. After the release of the occlusion, hemoglobin levels should return to baseline levels. Real-time measurements of arterial occlusion with our device demonstrate the expected physiological response (Figure 4a), which is consistent with similar PPG and NIRS studies [41,42,43,44]. During arterial occlusion, the optical density change was bigger for the channel with a larger optode separation, but there were no other significant differences between the three channels.

The second validation test was performed with the sensor applied to the left prefrontal cortex. During a breath-holding exercise (Figure 4b), the oxygenated hemoglobin concentration in the brain drops during the initial 5–10 s, followed by a recovery in oxygenation that goes above the baseline and returns to baseline levels after the breath-holding is over. This observation is consistent with a diving response and cerebral autoregulation to maintain brain oxygenation [37,39]. There were no substantial differences between the three channels. Signals measured with our device on the prefrontal cortex contain the PPG waveform, which could be used in future studies to improve brain oxygenation detection [45,46]. Nevertheless, our device detected the initial event of decreased cerebral oxygenation in real time without advanced algorithms.

### 3.2. Real-Time Assessment of Cognitive Activity in Group 1

**Figure 5:**
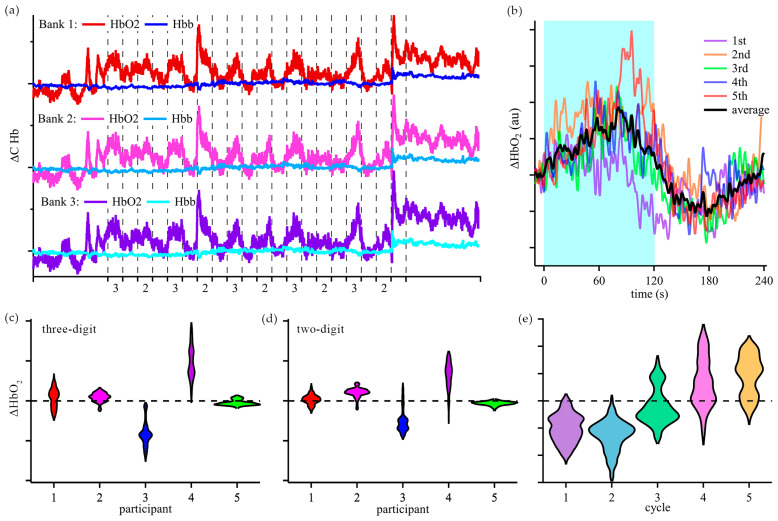
Time-domain fNIRS response to cognitive activity in Group 1. Three-digit and two-digit math operations were performed for 2 min with 2 min of rest for five cycles of each operation. (**a**) Sample participant data showing time-domain changes in hemoglobin concentrations. The time-domain fluctuations in oxygenated hemoglobin correspond to periods of cognitive activity. (**b**) Sample time-domain response to the three-digit arithmetic activity. The shaded region represents the 2-min arithmetic task, which is followed by 2 min of rest. Changes in oxyhemoglobin during 2 min of the three-digit (**c**) and two-digit (**d**) arithmetic tasks. (**e**) The oxy-hemoglobin response to the three-digit arithmetic task changed with the increasing number of cycles for one participant, correlating to fatigue.

Once the validation tests were concluded, changes in cerebral oxygenation during cognitive activities were assessed as participants were asked to perform arithmetic calculations with our device placed on the FP1 region. The typical time-domain response and calculated changes in hemoglobin concentrations of participants in Group 1 are shown in Figure 5. Group 1 was analyzed by evaluating the changes in oxygenated hemoglobin concentration during the arithmetic tasks in reference to the end of the previous resting period. Violin plots of concentration changes were created to visualize the average behavior over the five cycles of three-digit and two-digit math problems (Figure 5c,d). A unique trend was observed for participant 5, where the response to arithmetic task changed over the course of the experiment (Figure 5e).

### 3.3. Frequency–Domain Assessment of Cognitive Activity in Group 2

**Figure 6:**
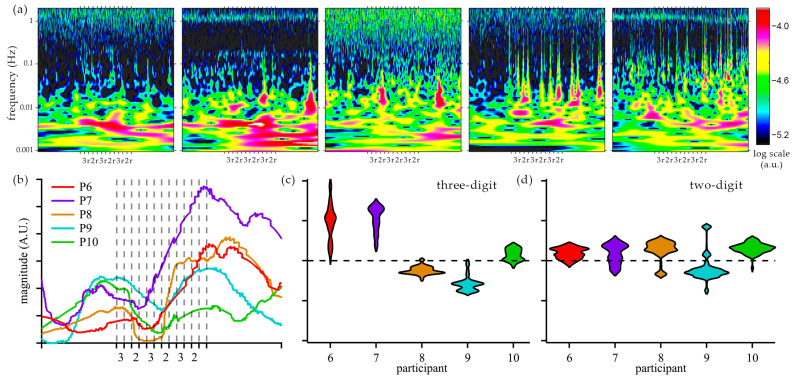
Analysis of oxy-hemoglobin changes in the participants in Group 2. (**a**) Wavelet transform of Homer3-processed oxy-hemoglobin changes during resting and arithmetic tasks for participants 6–10. (**b**) Synchrosqueezed magnitude of the 2 mHz component for participants in Group 2. Time-domain changes in oxygenated hemoglobin during 2 min of three-digit (**c**) and two-digit (**d**) arithmetic.

Raw sensor data was processed in Homer3 to reduce signal artifacts. Changes in oxy-hemoglobin were analyzed using a wavelet transform to obtain time–frequency responses for Group 2 participants (Figure 6a). The dominant components of the fNIRS signal are at a low frequency (below 40 mHz) for all participants in Group 2. Data for participants 7 and 10 exhibited cardiac oscillations around 1 Hz. However, no substantial data were observed in the time–frequency analysis in the mid-frequency band (0.1–1 Hz), where weak Mayer waves and respiratory signal may appear. Recorded signals were consistent with the literature results for fNIRS signals at FP1. Frequency components of interest were analyzed across the entire population of Group 2 and plotted using the synchrosqueezed transform, which improves the resolution of temporal changes [47]. Increased activity in a very low frequency (VLF) [48] of ~2.2 mHz was observed for participants 6, 7, and 8 after the first cycle of arithmetic tasks (see Figure 6b). Increased activity at 2.2 mHz was not observed for participants 9 and 10. In addition, Group 2 results were analyzed using the time-domain technique applied to Group 1 (see Figure 6c,d).

## 4. Discussion

During mental activity, an increase in cerebral oxygenation is expected based on prior fMRI and fNIRS studies [41,42,43,44]. While most of the participants in the Group 1 study exhibited an increase in oxygenation during the arithmetic tasks, the trend was not universal. Time-domain data from the participants can be classified into two sets based on the oxy-hemoglobin concentration (see Figure 5b). In one set, the oxy-hemoglobin level increased during arithmetic calculations (participants 1, 2, and 4). In the other set, the oxy-hemoglobin concentration decreased during arithmetic tasks (participant 3). In both sets, the changes in cerebral oxygenation for three-digit calculations were bigger than for two-digit calculations. Notably, participant 5 displayed characteristics of the second set for the first two cycles of the three-digit and two-digit calculations, and then displayed characteristics of the first set during cycles 4 and 5. This participant provided verbal answers to the arithmetic questions and demonstrated effort and mostly correct answers throughout the testing period. Based on the oxygenated hemoglobin changes during the cognitive tasks and observations, we speculate that the change in response is indicative of a change from effort to fatigue during the cognitive tasks. These results demonstrate the potential to monitor cognitive activity without significant post-processing, which is critical for edge computing.

The assessment of mental activity is inherently limited by the reliance on self-reporting or observed behavior. In the Group 1 study, some participants did not provide verbal answers to arithmetic problems, which limited our ability to correlate observed signals to the level of cognitive effort. One participant discontinued the arithmetic tasks midway through the study due to stress caused by the calculations; their incomplete data was excluded from the analysis. To promote recruitment and adherence, the participants were only encouraged to answer questions aloud. During the Group 1 study, the frequency of yawning increased in cycles 3 and 4 (see Supplementary Materials), which increased signal artifacts and may affect cerebral oxygenation. To minimize the effects of yawning, the Group 2 protocol was limited to three cycles of arithmetic tasks and included an extended rest period for frequency analysis.

The frequency analysis of Group 2 participants revealed significant contributions from frequencies below 40 mHz (Figure 6a). Specifically, for participants 6 and 7, the magnitude of VLF components increased during the cognitive tasks and persisted into the long resting period. For participants 6, 7, and 8, the synchrosqueezed analysis showed an increase in the 2.2 mHz component after the initial cycle of arithmetic tasks and a decrease during the long resting period, which is consistent with cognitive fatigue and recovery. Signals observed in the very low frequency (VLF) range [49,50] are sensitive to mental workload [48,51] and are not affected by other physiological signals, such as Mayer waves and cardiac and respiratory signals [48]. While our study block design has a frequency component around 2.1 mHz (8-min period), it would be low in amplitude and does not match the observed contributions, which last into the long resting period.

Frequency analysis of participants 9 and 10 revealed spikes that became more frequent with testing and correlated with observed yawning. The idea of yawning as a means of brain oxygenation recovery has been disputed [52,53,54]. However, it is possible that yawning increases heart rate, which could expel a higher volume of CO_2_ and increase oxygenation [55]. The effects of yawning on cognitive activity or fatigue were not incorporated in our study.

The frequency analysis of Group 2 separates the participants into two sets based on the VLF hemodynamic response: participants 6, 7, and 8, and participants 9 and 10. In addition, Group 2 participants were classified using the time-domain analysis of the three-digit and two-digit arithmetic tasks, similar to the Group 1 study (see Figure 6c,d). Participants 6 and 7 exhibited an increase in oxygenated hemoglobin during arithmetic tasks and a bigger response to three-digit tasks as compared to two-digit tasks. Participant 9 exhibited a decrease in oxygenated hemoglobin, with a more pronounced change for the more difficult task. The responses of participants 8 and 10 did not match the behavior observed in Group 1. The agreement between the time-domain and frequency-domain classification of participants engaged in the study demonstrates the potential of the developed system to measure cognitive activity. As our validation of cognitive effort and fatigue by participants is limited by oral responses during testing, it is possible that our device measured brain activity related to speech. Nevertheless, this work demonstrates the capabilities of the developed miniaturized device to capture hemodynamic changes correlated with cognitive activities.

The aim of our work was to advance portable sensors toward wearable and comfortable systems to enable medical monitoring during the activities of daily life. The developed device builds on previous advances in portable fNIRS devices by reducing the complexity of devices. Earlier developments in fNIRS devices have used flexible circuitry for wearable probe boards, with optical components connected to the main control board, which requires additional cables and hardware for data processing, affecting mobility and comfort [43,56,57,58]. More recent, compact designs utilize microcontrollers, FPGAs, and SoC components yet still have multiple parts connected via flexible cables [59,60], reducing comfort and practicality. A flexible and portable design has previously been demonstrated, but it lacked cognitive activity recognition and suffered from a short battery life [61]. Our device enables more than 50 h of hemodynamic recordings for unrestricted cognitive activity monitoring. Table 2 summarizes the development of fNIRS devices toward portable sensing.

## 5. Conclusions and Future Work

In this work, we presented the design and development of an efficient, compact, wireless, wearable, and portable NIRS device for cognitive brain activity monitoring. The functionality of the system was demonstrated in arterial occlusion, breath-holding, and arithmetic cognitive task experiments. The classification of cognitive activities performed during the experiment was demonstrated in the time and frequency domains for real-time and offline analysis. The design process of this fNIRS system targets the monitoring of brain activity in unrestricted and unsupervised daily life environments. Future work aims to translate our device’s architecture into a conformable circuit board system by replacing traditional PCB with an ultra-thin polymeric one to further improve user comfort and increase optode separation for deeper probing of the cerebral cortex.

## Figures and Tables

**Table 1:** Assessment of the optical coupling factor of the optode to skin in the wearable fNIRS device after the addition of the filter and the filling of void spaces.

Optode	Before		After		Improvement	
	CF_DC_	CF_AC_	CF_DC_	CF_AC_	DC	AC
20.2 mm 840 nm	548 ± 18	52 ± 7	4390 ± 180	500 ± 75	8.0×	9.7×
20.2 mm 660 nm	1060 ± 20	54 ± 10	11,830 ± 420	1190 ± 190	11.2×	22.0×
17.7 mm 840 nm	978 ± 33	52 ± 12	5430 ± 220	620 ± 73	5.6×	6.5×
17.7 mm 660 nm	1090 ± 20	53 ± 9.5	8430 ± 300	790 ± 130	7.8×	14.9×
15.2 mm 840 nm	1830 ± 70	200 ± 25	7100 ± 270	720 ± 100	3.9×	3.7×
15.2 mm 660 nm	3270 ± 50	140 ± 22	15,080 ± 460	1200 ± 190	4.6×	8.6×

**Table 2:** Comparison of portable fNIRS device studies and their demonstrated uses.

System Architecture	Intended Usage	Demonstrated	Wearability	Ref.
Probe board/control box/PC computer; two channels	Bedside hemodynamic motoring in neonatal patients	Brain oxygenation and blood volume measurements on neonatal patients	Not wearable and not wireless. For bedside assessment in clinics.	[27]
Probe Board/conv. board/FPGA processing; 48 configurable channels	Hemodynamic monitoring, brain mapping	No use cases; conceptual discussion	Not wearable and connected to an external FPGA development board.	[57]
Flexible probe board/ARM microprocessor main board; 48 channels	Hemodynamic monitoring, brain mapping	Breath-holding	Not wearable, probe board potentially uncomfortable, and multiple parts connected by a cable.	[62]
Headband sensor pad/PC computer processing unit; 16 channels	Hemodynamic monitoring, brain mapping, cognitive action recognition	Mental workload assessment measures, n-back test, and UAV flight simulators (24 participants)	Not wearable; mobility affected; and has multiple parts, including a PC connected with cables.	[58]
Headband sensor pad/Intel SoC control unit; 16 channels	Hemodynamic monitoring, brain mapping	Breath-holding	Not wearable, multiple parts, design not compact, very large probe board, and multiple cables to connect the modules.	[43]
Flexible probe board/FPGA controlled system/wireless data transmission; 18 channels	Hemodynamic monitoring, brain mapping	Brain mapping under workload stimuli (12 participants)	Not wearable; comfort issues; probe circuit, battery, and control unit on a wide headband.	[59]
Flex probe/AFE module /processor + BLE module; 6 channels	Hemodynamic monitoring, brain mapping	Hyperventilation, breath-holding, and motor tasks (1 participant)	Not wearable, comfort issues, and multiple parts attached to a headband.	[60]
Flexible circuit/onboard probing/BLE/ Nordic SoC nRF52832; 4 channels	Hemodynamic monitoring, brain oxygenation tracking on neonatal patients	Oxygenation measurements (SpO2) on subjects in a 2-month- to 15-year-old range	Wearable, flexible, and compact; short battery life of 1 h.	[61]
HEGduino/probing and processing boards/MAX86141 processor/BLE; 1 channel	Hemodynamic monitoring	Hemoglobin changes obtained while performing multiple tasks	Not wearable, comfort issues, multiple parts arranged in a huge headband.	[63]
MAX86141 processor/probing and processing boards/BLE; 2 channels	Hemodynamic monitoring	Real-world recording of non-social and social prospective memory tasks	Not wearable and has multiple cables around the body, connecting different modules and affecting comfort.	[64]
Silicon Labs SoC integrated into the probes and data-conversion components; 3 channels	Hemodynamic monitoring	Occlusion, breath-holding, and mental workload recognition (10 participants)	Wearable, single-part device fixed with medical tape; comfortable, mobility-compatible, and capable of multi-day readings.	This work

## Data Availability

The raw data supporting the conclusions of this article will be made available by the authors upon reasonable request.

## Supplementary Materials

The following supporting information can be downloaded at: https://www.mdpi.com/article/10.3390/bios15020092/s1, 1. Control UUID; 2. LED Safety Assessment; 3. Statistical Analysis Of Readings; 4. Optical Configuration; 5. Validation Test Setup; 6. Current Consumption and Battery Life; 7. Arithmetic Study Design; 8. Group 1 and 2 Results; Figure S1. Structure of the control UUID; Figure S2. Self-calibration routine for the fNIRS device. The device enters calibration mode when the command 0 × 1000 is sent over a BLE transmission. The self-calibration example was acquired with the sensor applied to a participant’s wrist, and photoplethysmography oscillations are visible in the signal after calibration; Figure S3. Assembly diagram of the LED on the PCB surface with the shroud around it: the opening area for the light is a 2 mm × 1 mm rectangular aperture. h is the height of the light emission point, which is 0.25 mm for the 660 nm LED and 0.28 mm for the 840 nm one; Figure S4. Distribution of the reading results for 5 samples per measurement; Figure S5. Detailed configuration of the fNIRS optical system. Drawing units are millimeters; Figure S6. Battery current consumption over time on standby; Figure S7. Battery current consumption over time when the sensor is connected and running measurements; Figure S8. Block protocol design for Groups 1 and 2; Figure S9. Overall hemoglobin changes for all 5 participants in Group 1. The results for participants 1, 3, and 5 show an overall oxygenation decrease. These three participants happened to be the ones with a higher number of yawns during the test performance; Figure S10. Yawns observed during the three-digit computation tests, which caused spikes in the data. Spikes appear in both oxy- and deoxygenated hemoglobin changes; Figure S11. Yawns observed during the two-digit computation tests; Figure S12. Group 2A’s results for oxy- and deoxy-hemoglobin concentration changes during the three-digit and two-digit mental math tasks. Participants 6 and 7 got the most consistent results in oxygenation increase; Figure S13. Group 2B’s results for oxy- and deoxyhemoglobin concentration changes during the three-digit and two-digit mental arithmetic tasks. No oxygenation increase was observed during the three-digit mental task performance. Delayed change in oxygenation may indicate mental fatigue; Table S1: Optical constants used to calculate the hemoglobin concentration changes; Table S2: Validation test settings (occlusion and breath holding); References [32,33,65,66,67,68] are cited in the Supplementary Materials.
