# Supplementary material for: Wearable Wireless Functional Near-Infrared Spectroscopy System for Cognitive Activity Monitoring
Source: Biosensors (Basel). 2025 Feb 6;15(2):92. doi: 10.3390/bios15020092 (PMC11853267; doi:10.3390/bios15020092)
Supplement: Supplementary file 1 [file biosensors-15-00092-s001.zip › biosensors-3429346-supplementary.pdf]

biosensors

MDPI

Supplementary Materials

Wearable Wireless Functional Near-Infrared Spectroscopy System for Cognitive Activity Monitoring

Mauro Victorio [1], James Dieffenderfer [2], Tanner Songkakul [2], Josh Willeke [3], Alper Bozkurt [2] and Vladimir A. Pozdin [1,4,*]

1 Department of Electrical and Computer Engineering, Florida International University, Miami, FL 33174, USA; maucabal@fiu.edu
2 Department of Electrical and Computer Engineering, North Carolina State University, Raleigh, NC 27695, USA; jpdieffe@gmail.com (J.D.); tsongkakul@gmail.com (T.S.); aybozkur@ncsu.edu (A.B.)
3 Department of Engineering Physics, Rose Hulman Institute of Technology, Terre Haute, IN 47803, USA; willekjw@rose-hulman.edu
4 Department of Mechanical and Materials Engineering, Florida International University, Miami, FL 33174, USA
* Correspondence: vpozdin@fiu.edu

Keywords: near-infrared spectroscopy (NIRS); functional NIRS (fNIRS); wearable devices; biosensors, brain activity; cognitive monitoring.

1. Control UUID

<table>
<tr><th colspan="8">Byte 0</th><th>Byte 1</th></tr>
<tr><th>MSB Bit 7</th><th>Bit 6</th><th>Bit 5</th><th>Bit 4</th><th>Bit 3</th><th>Bit 2</th><th>Bit 1</th><th>Bit 0 LSB</th><th>Gain / Intensity Value</th></tr>
<tr><td>0 - settings config.</td><td colspan="2">Number of Readings:
00 – No change
01 – 1 reading
10 – 3 readings
11 – 5 readings</td><td>Parameter:
0 – Gain
1 – Intensity</td><td colspan="2">Bank Definition:
00 – All banks
01 – Bank 1
10 – Bank 2
11 – Bank 3</td><td colspan="2">LED Definition:
00 – No change
01 – LED 1
10 – LED 2
11 – Both LEDs</td><td>Gain or intensity value, based on bit 4 on Byte 0, applied to the banks and LEDs defined in bits 3~0.

byte 0 = 0x10
byte 1 = 0x00 – self-calibration
byte 1 = 0x01 – measure background</td></tr>
<tr><td>1 - run Config.
.</td><td>Run Flag:
0 – Standby
1 – Run</td><td colspan="3">Bank Selection:
000 – No banks
001 – Bank 1
010 – Bank 2
011 – Bank 3
100 – Banks 1 & 2
101 – Banks 1 & 3
110 – Banks 2 & 3
111 - All banks</td><td colspan="2">LED Selection:
00 – No LED in bank
01 – LED 1
10 – LED 2
11 – Both LEDs</td><td>Sampling Freq.:
0 - 10Hz
1 - 20Hz</td><td>Not used</td></tr>
</table>

Figure S1. Structure of the control UUID.

The communication protocol developed to define the device operation enables flexible configuration, supporting parameters for the number of readings per data acquisitions (selectable as one, three, or five samples), the frequency of the measurement cycles (10 Hz or 20 Hz), the light intensity for each LED in each bank (adjustable by setting the driving current), and the gain applied to the ADC signal. Additionally, users can specify which LEDs to activate, allowing targeted measurements from specific banks. Commands are transmitted as two-byte words from the smart device connected to the sensor based on the control UUID format shown in Figure S1.

1.1. *Self-calibration algorithm*

To accommodate various skin complexions and variable ambient light environments, the sensor offers a self-calibration routine triggered by sending 0x1000 over the BLE connection. During self-calibration, the data measurement procedure continues

uninterrupted to maintain the session's timing. However, specific adjustments are made: the gain is temporarily set to 1, and the LED intensity is reset to 0. The LED intensity is individually incremented at each reading cycle until the signal reaches 100mV across the resistor in series with the SiPM detector. Once this condition is achieved, the gain is adjusted to bring the ADC readings to approximately 1000 units.

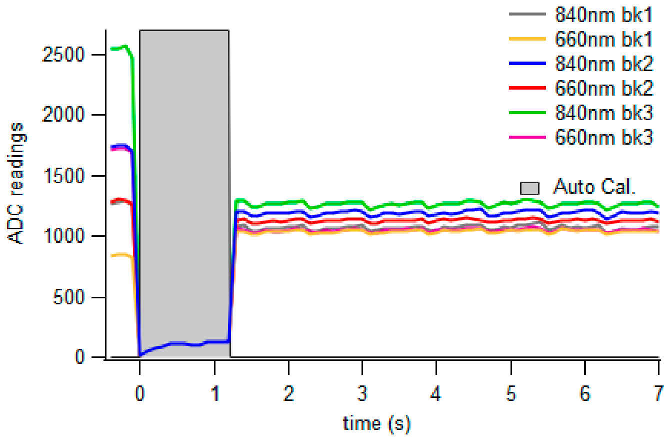

Figure S2. Self-calibration routine for the fNIRS device. The device enters calibration mode when the command 0x1000 is sent over the BLE. The self-calibration example was acquired with the sensor applied to a participant's wrist; photoplethysmography oscillations are visible in the signal after calibration.

2. LED Safety Assessment

Safe LED drive conditions for the fNIRS device were determined based on the IEC 62471 – Photobiological safety of lamps and lamp systems [1]. These standards are widely adopted in many countries, beyond the European Union. While the FDA has yet to publish specific documentation on this topic, IEC 62471 serves as an excellent guide for the American market as well.

The exposure limits in the IEC 62471 standard applicable to the fNIRS sensor include: (a) the retinal thermal hazard exposure limit, (b) the infrared radiation hazard exposure limits for the eye, and (c) the thermal hazard exposure limit for the skin. These criteria are detailed in Sections 2.1 to 2.3.

The device was designed for use on the forehead, in direct contact with the patient's skin with no direct eye exposure. However, for safety assessment purposes, we considered potential eye exposure when the sensor is being moved into its intended position and inadvertently directs light into the user's eye. In this scenario, a 10 mm distance is assumed, representing the distance from the device to the ocular globe, and a 10-second exposure time is considered.

2.1 Retinal thermal hazard exposure limit

The acceptable limits to avoid retinal thermal injury are defined by the spectral radiance of the light source, weighted by a burning function based on wavelength for the 660 nm source:

$$L_R = \sum_{380}^{1400} L_\lambda \, R(\lambda) \Delta\lambda \quad \leq \quad \frac{50000}{\alpha \cdot t^{0.25}} \qquad [\mathrm{W} \cdot m^{-2} \cdot \mathrm{sr}^{-1}] \qquad (1)$$

where

L_R is the integrated spectral radiance

L_λ is the spectral radiance [$\mathrm{W{\cdot}m^{-2}{\cdot}sr^{-1}{\cdot}nm^{-1}}$]

$R(\lambda)$ is the burn hazard weight function

$\Delta\lambda$ is the bandwidth in nm

α is the angular subtense of the light source in radians

The conservative limit to avoid retinal thermal injury due to the 840 nm source is given by the weak visual stimulus limit:

$$L_R = \sum_{780}^{1400} L_\lambda R(\lambda)\Delta\lambda \quad \leq \frac{6000}{\alpha} \quad [\mathrm{W} \cdot m^{-2} \cdot \mathrm{sr}^{-1}] \tag{2}$$

The spectral radiance is given by

$$L_\lambda = \frac{d\phi(\lambda)}{dA{\cdot}cos\theta{\cdot}d\Omega{\cdot}d\lambda} \quad [\mathrm{W} \cdot m^{-2} \cdot \mathrm{sr}^{-1} nm^{-1}] \tag{3}$$

where

$d\phi(\lambda)$ is the spectral radiant power

θ indicates the angle between the position vector from the light source point to the area element and the perpendicular vector to the area surface element. $\theta = 0°$ was used

$dA \cdot cos\theta$ is the area element times the $cos\theta$

$d\Omega$ is the solid angle, given in sr

$d\lambda$ is the bandwidth in nm

The geometry of the LED assembly on the board surface, with the shroud-defined aperture, is shown below.

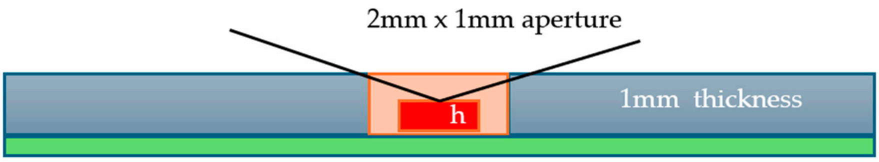

Figure S3. Assembly diagram of the LED on the PCB surface with the shroud around it; the opening area for the light is a 2 mm x 1 mm rectangular aperture. h is the height of the light emission point, which is 0.25 mm for the 660 nm LED and 0.28 mm for the 840 nm one.

The solid angle, Ω, is given by [2]

$$\Omega = 4sin^{-1}\left\{\frac{lb}{\sqrt{(l^2+4d^2)\cdot(b^2+4d^2)}}\right\} \tag{4}$$

where $d = 1\ mm - h$, and l and b are the side dimensions of the aperture (2 mm x 1 mm)

The values calculated for both wavelengths are as follows:

$$\Omega_{660nm} = 1.84\ \mathrm{sr},\ \ \Omega_{840nm} = 1.93\ \mathrm{sr} \tag{5}$$

These solid angle values are more than 40% smaller than the solid angles calculated from the half angle in the manufacturer's LED specifications. Solid angle values from equation 5 were utilized to calculate conservative limits, as a smaller area results in greater irradiance power.

The surface area of the solid angle formed from the light source to the pupils will be the following:

$$\mathrm{A}_{660nm} = \Omega_{660nm} \times \mathrm{r}^2 = 1.84 \times (0.01)^2 = 1.84 \times 10^{-4}\ \ m^2 \tag{6}$$

$$\mathrm{A}_{840nm} = \Omega_{840nm} \times \mathrm{r}^2 = 1.93 \times (0.01)^2 = 1.93 \times 10^{-4}\ \ m^2 \tag{7}$$

Based on the LED characterization, we tested the maximum total irradiance at 30 mA drive current, which corresponds to

$$\mathrm{P}_{\mathrm{660nm,30mA}} = 2.42\ \mathrm{mW},\ \ \mathrm{P}_{840nm,30mA} = 12.16\mathrm{mW} \tag{8}$$

To determine the exposure to the retina, the following were considered: (1) the sensor is placed 10 mm away from the eye, (2) the LED is on the entire time, and (3) the exposure condition is 10 seconds. To define the area through which light passes to reach the retina, the subtense angle α is considered. This is the angle from the light source to the measuring point, which is the user's eye in this case [1]. For our geometry, the maximum angle of 0.1 rad was adopted per the standard. Retinal hazard limits are calculated below.

$$L_{R_{660nm}} = \frac{2.42 \times 10^{-3}\ W}{1.84\ \times 10^{-4}\text{m}^2\ \times 1.84\text{sr}} \times 1 = \ 7.15\ Wm^{-2}sr^{-1} \ll 2.8 \times 10^5\ \ Wm^{-2}sr^{-1} \quad (9)$$

$$L_{R_{840nm}} = \frac{12.16 \times 10^{-3}W}{1.93\ \times 10^{-4}\ \text{m}^2 \times 1.93\text{sr}} \times 0.525 = \ 17.1\ Wm^{-2}sr^{-1} \ll 6 \times 10^4\ Wm^{-2}sr^{-1} \quad (10)$$

The maximum irradiance values for our devices are orders of magnitude below the retinal hazard limits, even with conservative estimates.

2.2 Infrared radiation hazard exposure limits for the eye

In this section, we calculate the spectral irradiance limit of the device to prevent thermal injuries to the cornea and the ocular lens of the eye (such as cataract formation) caused by infrared radiation in the 780 to 3000 nm wavelength range. For exposure times under 1000 seconds, which apply to our device, the exposure level limits are given by the following:

$$E_{IR} = \sum_{780}^{3000} E_\lambda \cdot \Delta\lambda \quad \le \ 18000 \cdot t^{-0.75} \quad (11)$$

where

E_{IR} is the total infrared irradiance [W·m^{-2}]

E_λ is the spectral irradiance [W·m^{-2}·nm^{-1}]

The spectral radiant power limit is as follows:

$$E_{IR} = \frac{12.16 \times 10^{-3}W}{1.93\ \times 10^{-4}\ m^2} = 63\ W/m^2 \le 18000 \times (10)^{-0.75} = 3.2\ \times 10^3 W\ \ m^{-2} \quad (12)$$

The 840 nm radiant power is below the safety limit by orders of magnitude.

2.3 Thermal hazard exposure limit for the skin

The thermal injury exposure limit for the skin in the visible and infrared range (380 nm to 3000 nm) is defined by

$$E_H \cdot t = \sum_{380}^{3000} \sum_{t} E_\lambda(\lambda, t) \cdot \Delta t \cdot \Delta\lambda \quad \le \ 20000 \cdot t^{0.25} \qquad [J \cdot m^{-2}] \quad (13)$$

where

$E_\lambda(\lambda, t)$ is the spectral irradiance [W m^{-2} nm^{-1}]

With the device in contact with the skin on the forehead and neglecting optical losses, the power spectral irradiance is fully transferred to the skin through the aperture (1 mm by 2 mm). For the worst-case scenario, we calculate 10-second exposure to each LED:

$$E_{H660nm} \cdot t = \frac{2.42\ mW}{2\ mm^2} \times 10\ s\ = 1.21 \times 10^4\ J/m^2\ < 3.56 \times 10^4\ J/m^2 \quad (14)$$

$$E_{H840nm} \cdot t = \frac{12.16\ mW}{2\ mm^2} \times 10\ s\ = 6.08\ \times 10^4 J/m^2 > 3.56 \times 10^4\ J/m^2 \quad (15)$$

For the worst-case scenario, exposure to a 660 nm LED does not exceed the safety limit, while 840 nm exposure does. Based on the conservative calculations, the limit for 840 nm LED should be 7.1 mW at 18 mA for 10 seconds of continuous exposure. Software safeguards need to be added to limit accidental continuous exposure to prevent the thermal injury limit from being reached. EIC 62471 does not provide guidance for exposure

greater than 10 seconds, and prolonged continuous light exposure must be controlled based on the heat stress caused to the skin [3,4].

The thermal exposure limit under the normal device operation limit was calculated with 1.1 ms exposure for each LED (the maximum duration with the five-readings mode) at a 20 Hz sampling rate, which indicates that the light is active 2.2% of the time. Even with six channels active, 86.8% of the time, there is no light emission from any LED, which is considered sufficient for skin recovery, not causing any heat discomfort for the user. Nevertheless, under normal operation, the energy dose applied to the skin must be below the limit for the exposure time the light is on:

$$E_{H_{660nm}} \cdot t = \frac{2.42mW}{2mm^2} \times 1.1ms = 1.3\,J/m^2 \ll 20000 \cdot t^{0.25} = 3.6 \times 10^3 J/m^2 \quad (16)$$

$$E_{H_{840nm}} \cdot t = \frac{12.16mW}{2mm^2} \times 1.1ms = 6.7\,J/m^2 \ll 3.6 \times 10^3\,J/m^2 \quad (17)$$

Regarding skin exposure, the dosage imposed by the LEDs is orders of magnitude lower than the safety limits. The calculations presented in this section indicate that the sensor is within the safety limits in all three applicable hazards, which are the thermal hazard to the retina, the infrared radiation hazard for the eye, and the skin exposure hazard.

3. Statistical Analysis of ADC Readings

The test setup for the statistical analysis of multiple readings was made by positioning the sensor inside a closed box to avoid external luminous interference; a reflective surface was used to provide the path between the light source and the photodetector. The device ran for 30 seconds, obtaining 300 samples per reading (1 to 5). The amplifier gain was set to 1 and the LED's current intensities were defined to reach 100 mV for the ADC input. Pairwise t-tests with a 1% significance level were applied to evaluate whether the optical measurement reached a steady state. Five samples of each LED for each bank were evaluated (Figure S4).

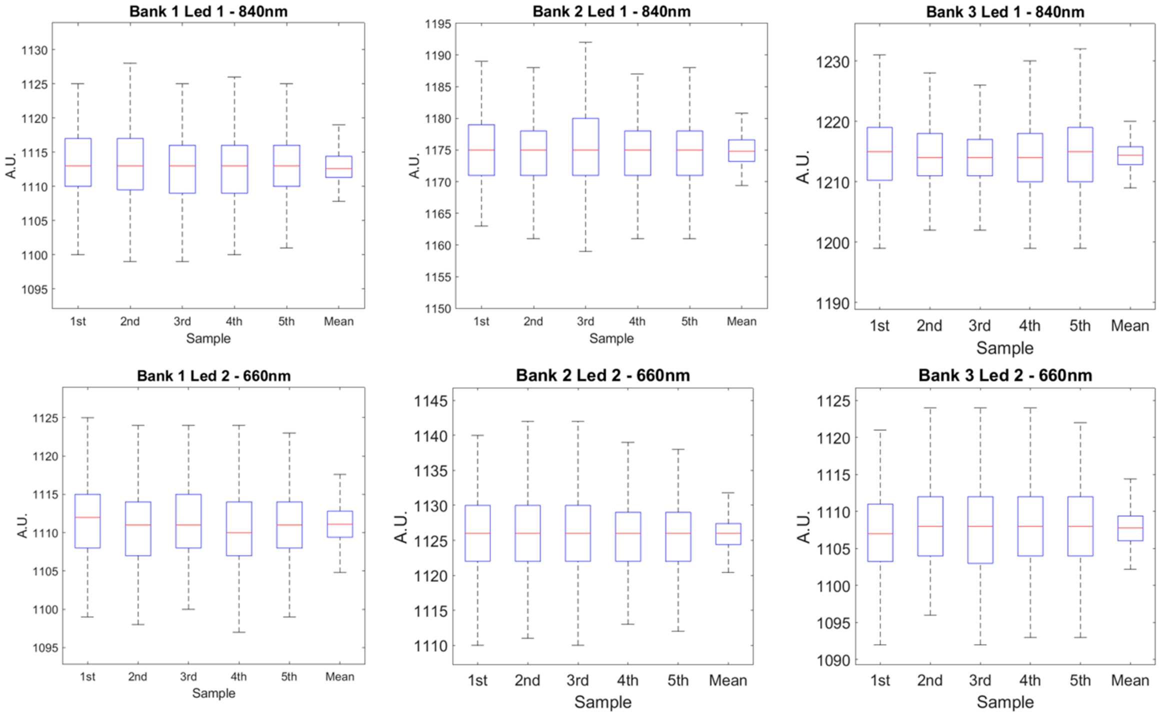

Figure S4. Distribution of the reading results for 5 samples per measurement.

The results of t-test for each LED are presented in a matrix format, where each element of the matrix is the comparison result of two data vectors according to their position indexes, comparing each sampling with all the other ones. There is one matrix per LED.

$$A_{BxLy} = \begin{bmatrix} a_{11} & \cdots & a_{15} \\ a_{21} & \cdots & a_{25} \\ \vdots & \ddots & \vdots \\ a_{51} & \cdots & a_{55} \end{bmatrix} \quad (18)$$

where a_{ij} is the t-test result from the *i* turn and *j* turn readings, $i, j = 1, 2, 3, 4, 5$.

where A_{BxLy} = matrix results for the t-test with Bank x and LED y components. Bank 1 is the 20.2 mm distance from the photodiode, and bank 2 and 3 are the 17.7 mm and 15.2 mm distance, respectively. In each bank, LED 1 is the 840 nm wavelength and LED 2 is the 660 nm wavelength.

Using a 800 µs time interval between light activation and data collection gives the results $A_{BxLy} = 0$, and the distribution results are presented in Figure S4. The results indicate that using the 800 µs time interval between light activation and data collection gives a stable response, with low oscillation or noise disturbances. Even so, acquisition can be enhanced by using average filtering (mean distribution in Figure S4).

4. Optical Configuration

The detailed geometry of the light sources and detector is shown in Figure S5.

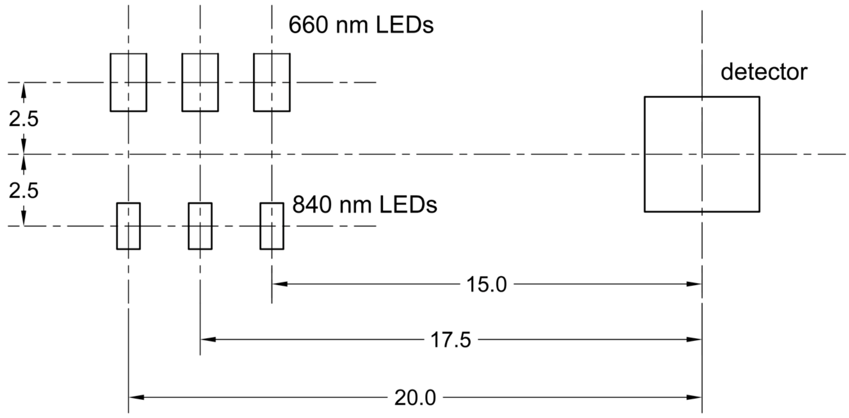

Figure S5. Detailed configuration of the fNIRS optical system. Drawing units are millimeters.

The constants used for the calculation of hemoglobin changes from optical density were obtained from the literature [5,6] and are summarized in Table S1.

Table S1. Optical constants used to calculate the hemoglobin concentration changes.

Constant	Description	Wavelength λ	Value
$\varepsilon_{HbO\lambda}$	Extinction coefficient (oxyhemoglobin)	660 nm	0.03346 $(mM.mm)^{-1}$
		840 nm	0.11072 $(mM.mm)^{-1}$
$\varepsilon_{Hb\lambda}$	Extinction coefficient (deoxyhemoglobin)	660 nm	0.34408 $(mM.mm)^{-1}$
		840 nm	0.07815 $(mM.mm)^{-1}$
$\mu_{a,\lambda}$	Absorption coefficient	660 nm	0.04944 mm^{-1}
		840 nm	0.03157 mm^{-1}
$\mu'_{s,\lambda}$	Scattering coefficient	660 nm	2.8845 mm^{-1}
		840 nm	2.2595 mm^{-1}

5. Validation Test Setup

The autocalibration routine was performed before running both the occlusion and the breath-holding tests, setting the LED intensity levels to match the optimal detection range of the SiPM photodetector. Table S2 shows the intensity and gain values for each optode channel.

Table S2. Validation test settings (occlusion and breath holding).

Test	LED	Intensity Current (mA)	Gain
Occlusion	Bk1 660 nm	16.73	10.77
	Bk1 840 nm	15.80	10.77
	Bk2 660 nm	25.16	10.77
	Bk2 840 nm	7.37	10.77
	Bk3 660 nm	14.62	10.77
	Bk3 840 nm	4.33	10.77
Breath Holding	Bk1 660 nm	7.37	10.77
	Bk1 840 nm	9.71	10.77
	Bk2 660 nm	15.33	10.77
	Bk2 840 nm	5.50	10.77
	Bk3 660 nm	9.48	10.77
	Bk3 840 nm	3.86	10.77

6. Current Consumption and Battery Life

Battery life was estimated by analyzing the current draw under different modes. In standby mode (Figure S5), the device advertises on the BLE, and the average battery current consumption is 5 mA (30 mA peak). Using a 500 mAh battery, the device can advertise for 100 h.

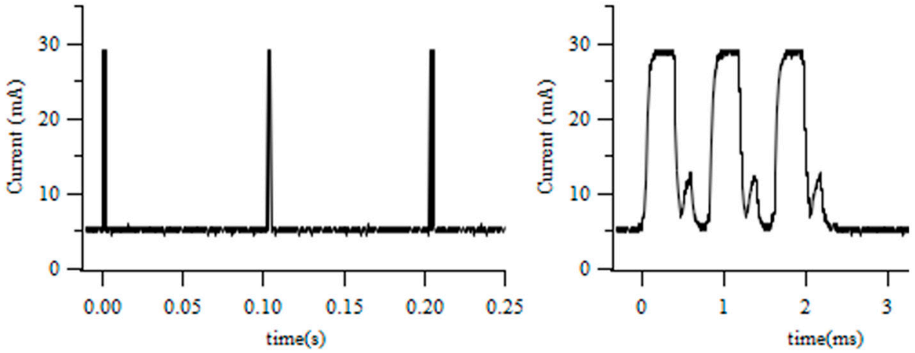

Figure S6. Battery current consumption over time on standby.

In operating mode (Figure S7), the average current consumption increases to 10 mA, with 70 mA peaks during optical measurements.

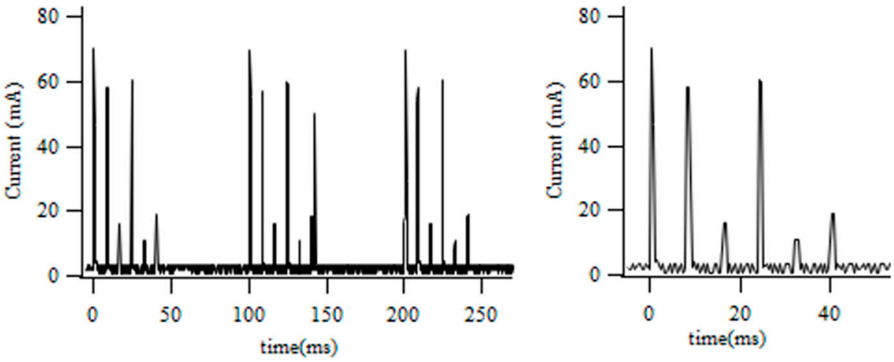

Figure S7. Battery current consumption over time when the sensor is connected and running measurements. Battery consumption of BLE transmission is shown at around 140 ms.

The 10 mA average consumption gives a battery life of 50 hours for a 500 mAh battery. The battery life can be extended by using non-continuous measurements or reducing the number of used channels. Enabling the deep sleep features on the BGM121 microcontroller can provide further reduction in power consumption and extend device operation.

7. Arithmetic Study Design

In this work, participants were in a seated position for the duration of the study. After verbal instructions from the study organizers, the participants watched a computer monitor during rest and for mental activities. Each session was designed with three uninterrupted, consecutive tasks: i) baseline resting, ii) cyclic mental tasks, and iii) relaxed recovery. For the baseline and recovery periods, the participants were shown videos from a playlist for a set duration of time. The videos included in the playlist are the following:

1) https://youtu.be/M1F0lBnsnkE?si=DtVFdCkK6_4XYk9H
2) https://youtu.be/dWVCYAdjS5M?si=6jswLnm-Ri_yRjWI
3) https://youtu.be/swloMVFALXw?si=c7kEsJsNBAMW26HI
4) https://youtu.be/9jIbvtD1cWw?si=oh9Qmh-Yk9PEyZrm
5) https://youtu.be/c08wiEyVuak?si=rlzPEvkwQGxfWXcF
6) https://youtu.be/LOILZ_D3aRg?si=Pcga9zgIIctDnp0u
7) https://youtu.be/LOILZ_D3aRg?si=5VFC-M85BM7hupNF
8) https://youtu.be/TjJHnKw7YNA?si=Zc58GVvEoMOXyovl
9) https://youtu.be/TjJHnKw7YNA?si=bI_b50xQ6yDtNyk7
10) https://youtu.be/oWFPBsP0exo?si=ENj0XKYIFNI4A__Y
11) https://youtu.be/F2zTd_YwTvo?si=EzPFNB282dxtBvfD
12) https://youtu.be/0rLhJZTHYo4?si=3IVPAigcd72x1sqQ

The video sequence was chosen considering that no emotions are intended to be elicited in the participants. A neutral condition recording is the goal of the baseline task, while keeping the individuals awake and alert during the initial part of the test, to provide meaningful data for the baseline. Based on these objectives, this video sequence was chosen to include dancing excerpts from several musical movies.

For cognitive fatigue assessment, the study participants were asked to perform a series of two-digit and three-digit two-number additions. During the task, participants were shown math additions for an interval of 3 seconds for a duration of 2 minutes. The participants were instructed to answer the questions aloud. Research staff did not interrupt the task if the participant did not answer the questions aloud and noted participants' participation and effort in arithmetic tasks. During the 2 minutes between arithmetic tasks, a countdown timer was shown to the participants.

In our work, the study was conducted in two groups, as illustrated in Figure S7. The study design was modified to increase the resting period and allow for longer baseline

and recovery periods. In addition, the number of math cycles was reduced from five to three due to the observed mental strain displayed by the initial participants.

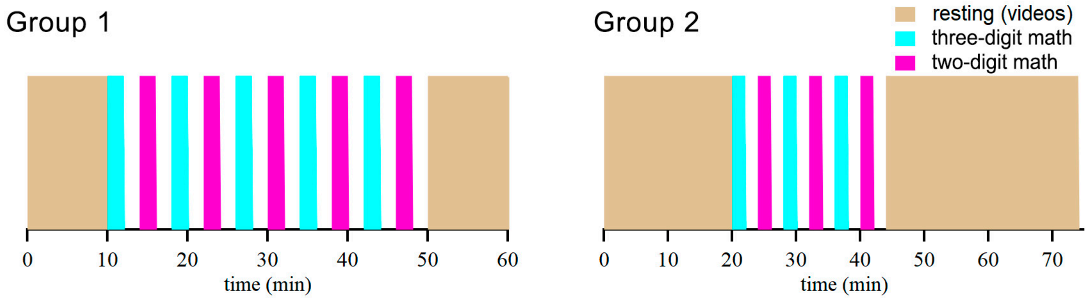

Figure S8. Block protocol design for Groups 1 and 2.

8. Groups 1 and 2 Results

The hemoglobin changes during the mental tasks in Group 1 are shown in Figure S9.

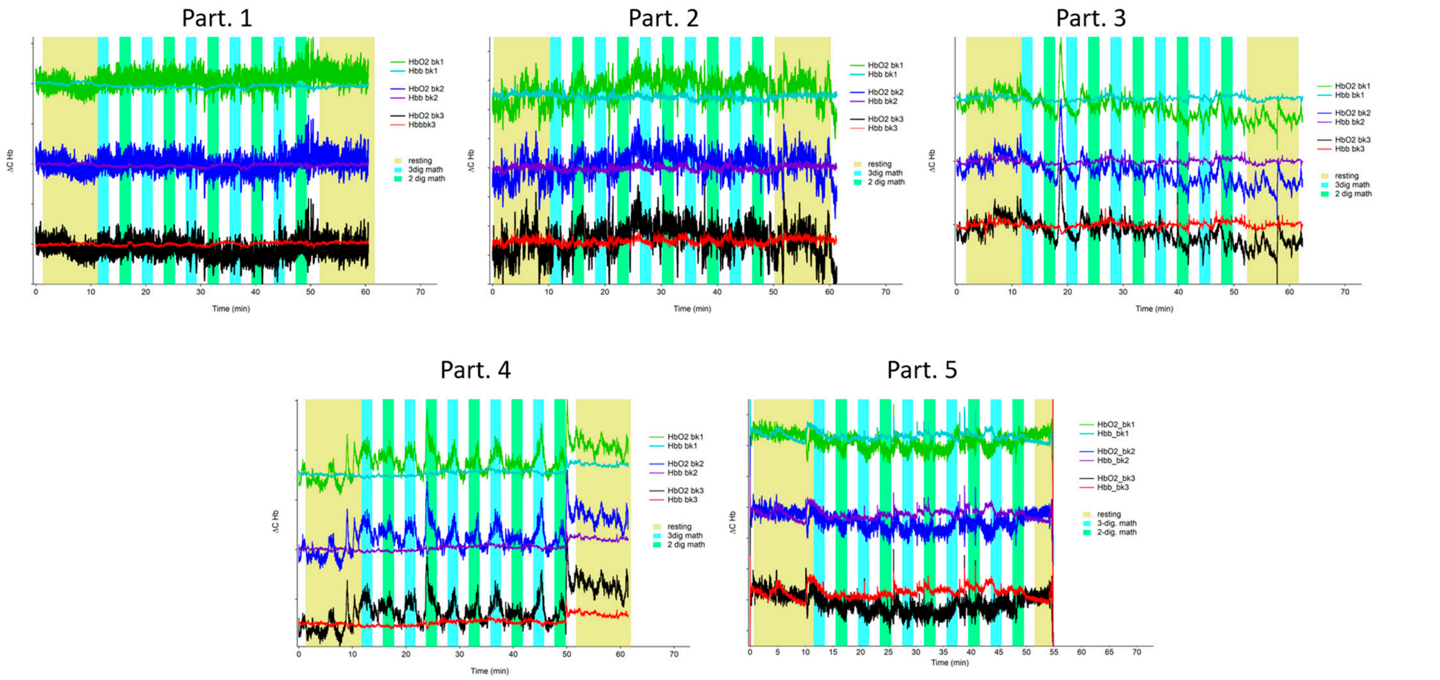

Figure S9. Overall hemoglobin changes for all 5 participants in Group 1. The results for participants 1, 3, and 5 show an overall oxygenation decrease. These three participants happened to be the ones with a higher number of yawns during the test performance.

The observation notes taken by the staff members conducting and supervising the tests indicate that those participants had several yawning events during the test, Figure S10-10.

The number of observed yawns was reduced in Group 2, as compared to Group 1. Figures S11 and S12 show the annotated yawns during Group 2 arithmetic tasks.

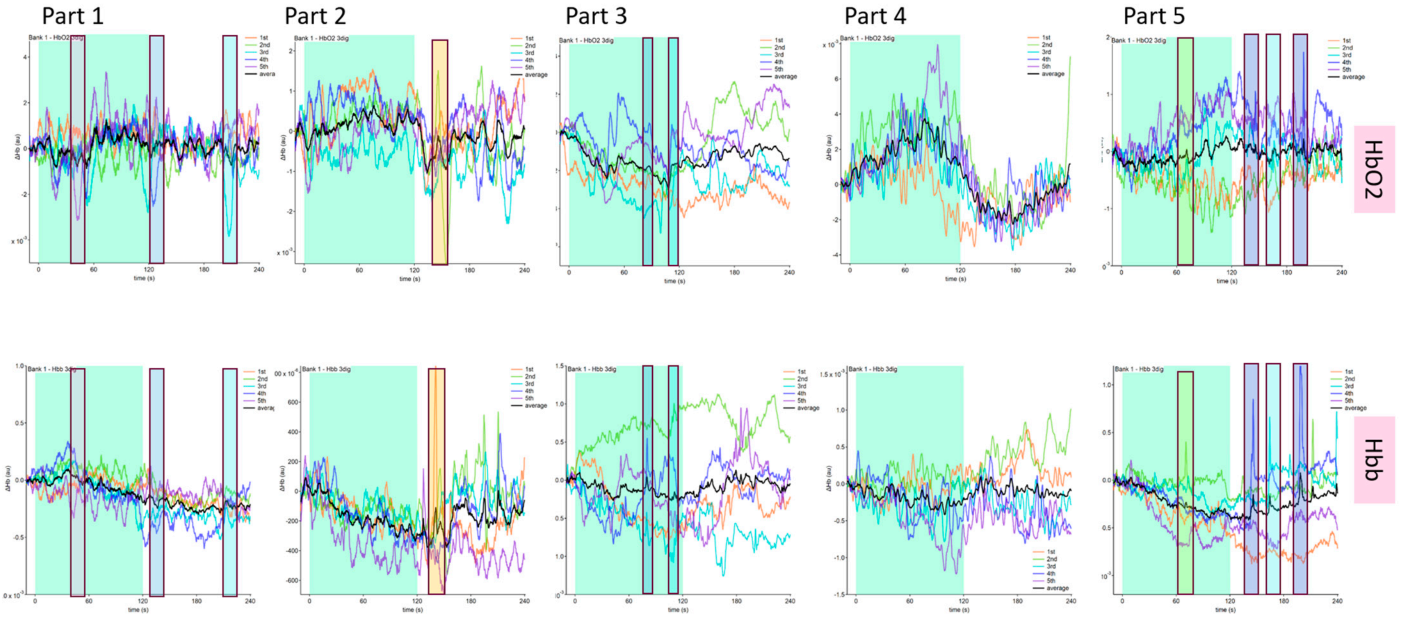

Figure S10. Yawns observed during the three-digit computation tests, which caused spikes in the data. Spikes appear in both oxy- or deoxygenated hemoglobin changes.

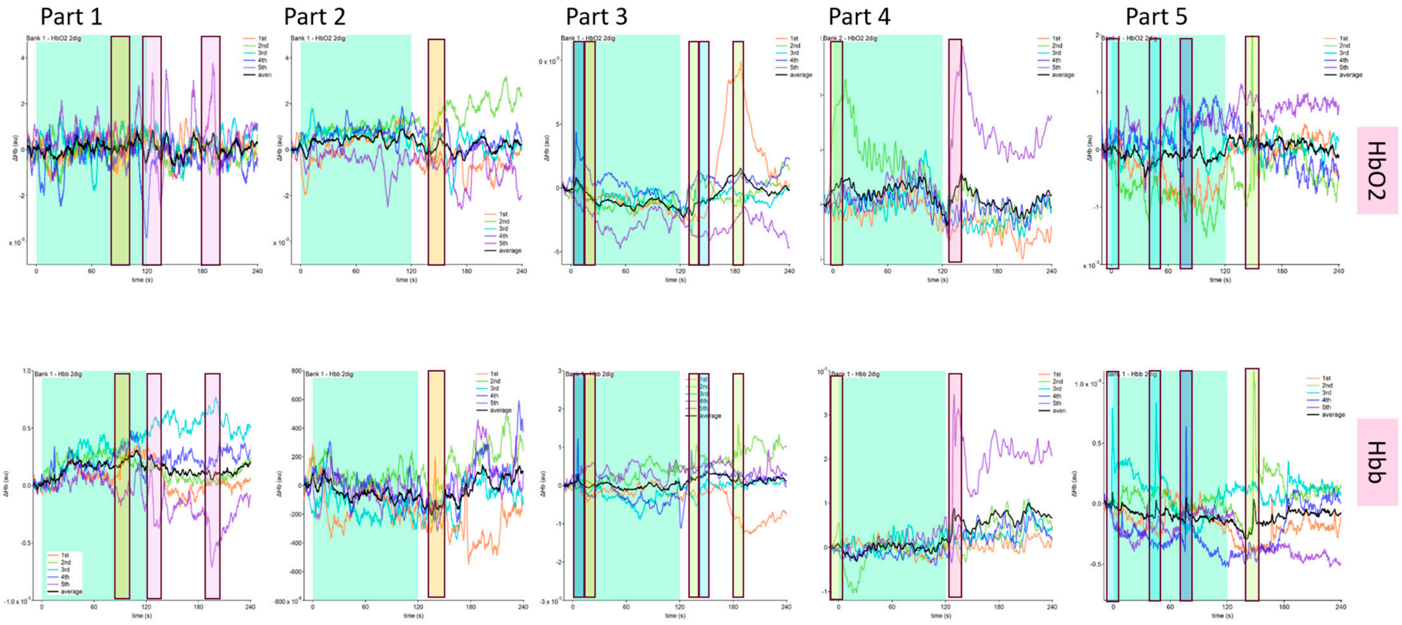

Figure S11. Yawns observed during the two-digit computation tests.

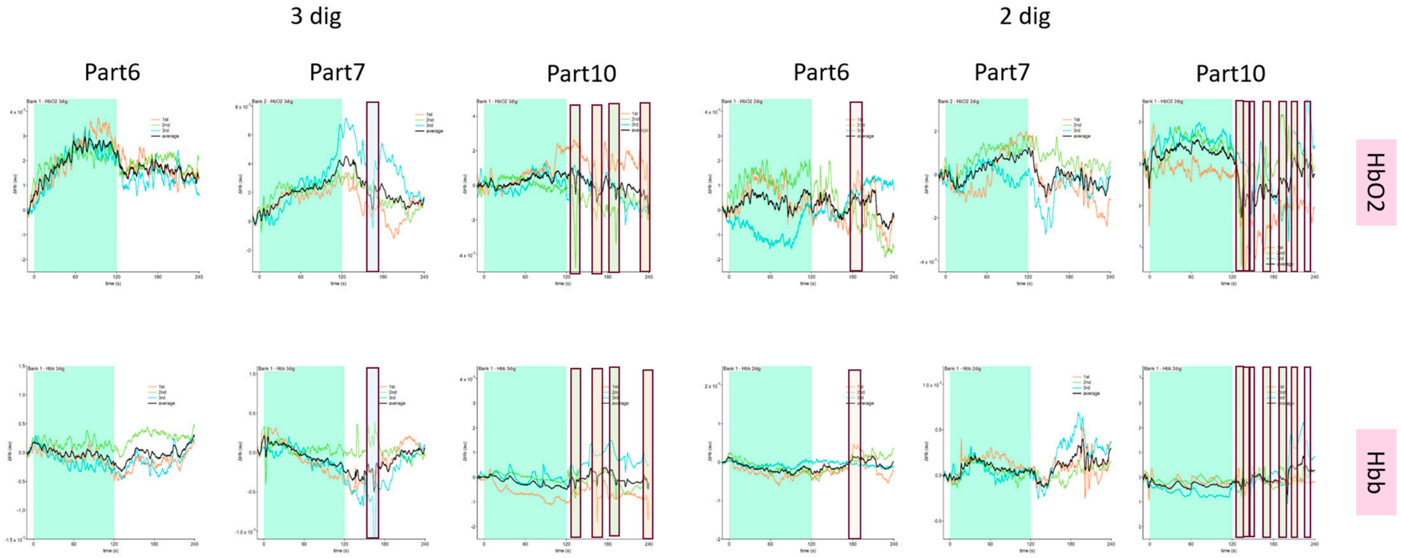

Figure S12. Group 2A results for oxy- and deoxyhemoglobin concentration changes during three-digit and two-digit mental arithmetic tasks. Participants 6 and 7 obtained the most consistent results of oxygenation increase.

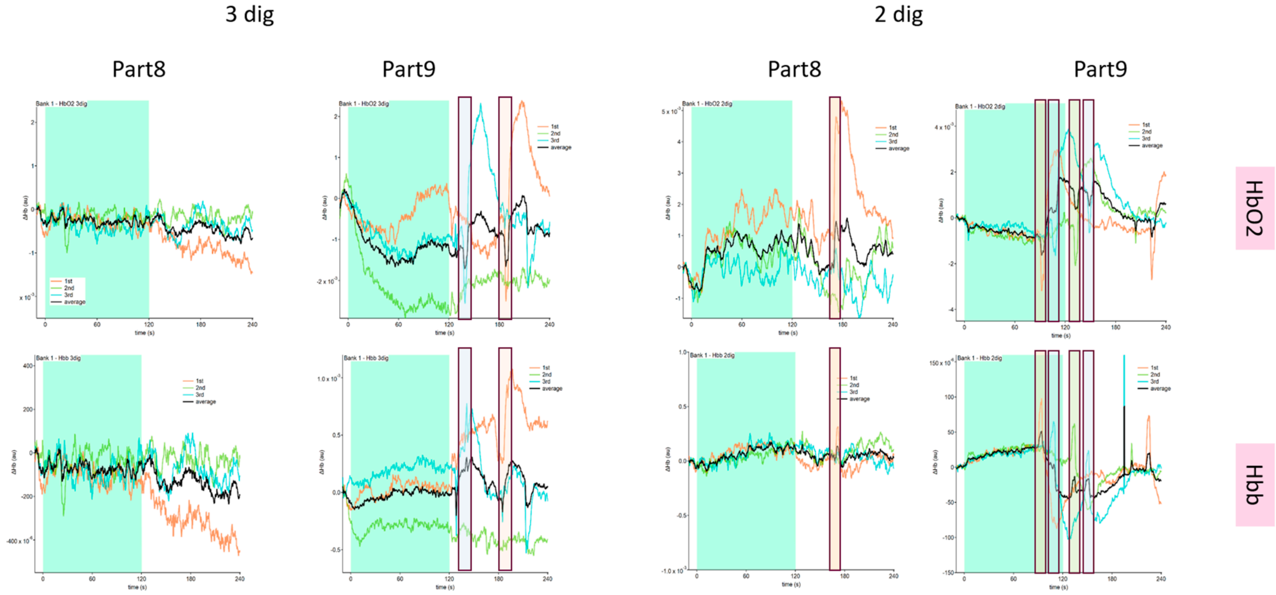

Figure S13. Group 2B results for oxy- and deoxyhemoglobin concentration changes during three-digit and two-digit mental arithmetic tasks. No oxygenation increase was observed during the three-digit mental task performance. Delayed change in oxygenation may indicate mental fatigue.

References

1. Commission, I. Photobiological safety of lamps and lamp systems. *IEC Geneva* **2006**, *62471*, doi:10.25039/S009.2002.
2. Rajpoot, H.C. HCR's Theory of Polygon (proposed by harish chandra rajpoot) solid angle subtended by any polygonal plane at any point in the space. *Int. J. Math. Phys. Sci. Res* **2014**, *2*, 28-56.
3. Protection, I.C.o.N.-I.R. ICNIRP guidelines on limits of exposure to incoherent visible and infrared radiation. *Health Physics* **2013**, *105*, 74-96, doi: 10.1097/HP.0b013e318289a611.
4. Kourkoumelis, N.; Tzaphlidou, M. Eye Safety Related to Near Infrared Radiation Exposure to Biometric Devices. *The Scientific World Journal* **2011**, *11*, 902610, doi:10.1100/tsw.2011.52.

5. Cope, M. *The development of a near infrared spectroscopy system and its application for non invasive monitoring of cerebral blood and tissue oxygenation in the newborn infants*; University of London, University College London (United Kingdom): 1991.
6. Van der Zee, P.; Essenpreis, M.; Delpy, D.T. Optical properties of brain tissue. In Proceedings of the Photon Migration and Imaging in Random Media and Tissues, 1993; pp. 454-465.

Disclaimer/Publisher's Note: The statements, opinions and data contained in all publications are solely those of the individual author(s) and contributor(s) and not of MDPI and/or the editor(s). MDPI and/or the editor(s) disclaim responsibility for any injury to people or property resulting from any ideas, methods, instructions or products referred to in the content.
